# Electrokinetic Remediation of Zn-Polluted Soft Clay Using a Novel Electrolyte Chamber Configuration

**DOI:** 10.3390/toxics11030263

**Published:** 2023-03-12

**Authors:** Zhaohua Sun, Wanxia Tan, Jian Gong, Guowei Wei

**Affiliations:** 1 School of Transportation and Civil Engineering, Nantong University, Nantong 226019, China; 2 Key Laboratory of New Technology for Construction of Cities in Mountain Area, Ministry of Education, Chongqing University, Chongqing 400045, China; 3 Guangxi Key Laboratory of Disaster Prevention and Engineering Safety, Guangxi University, Nanning 530004, China; 4 Jiangsu Zhongnan Construction Industry Group Co., Ltd., Nantong 226199, China

**Keywords:** electrokinetic remediation, fine-grained soil, heavy metal, electrolyte, configuration, electrokinetic geosynthetics

## Abstract

This study investigated a novel electrolyte chamber configuration for heavy-metal-contaminated fine-grained soil to reduce the leakage of electrolyte solution and alleviate secondary pollution, finally promoting the electrokinetic remediation (EKR) potential to be scaled up for application. Experiments were conducted on clay spiked with Zn to investigate the feasibility of the novel EKR configuration and the effect of different electrolyte compositions on the electrokinetic remedial efficiency. The results show that the electrolyte chamber situated above the soil surface is promising for the remediation of Zn-contaminated soft clay. Using 0.2 M citric acid as the anolytes and catholytes was an excellent choice for pH control in the soil and the electrolytes. Through this, the removal efficiency in different soil sections was relatively uniform and more than 90% of the initial Zn was removed. The supplementing of electrolytes resulted in the water content in the soil being distributed evenly and finally sustained at approximately 43%. Consequently, this study proved that the novel EKR configuration is suitable for fine-grained soil contaminated with Zn.

## 1. Introduction

Remediation of contaminated soil has important implications for environmental protection and the sustainable development of the economy. Electrokinetic remediation (EKR) is an efficient technique for the removal of inorganic and organic contaminants from fine-grained soil with minimal soil disruption [1,2,3,4,5,6]. It has been successfully applied in some actual cleanup projects to remove toxic heavy metals from soils [7,8,9]. However, several challenges have led to its limited implementation in the field over the past years. Due to the action of electroosmotic flow and electromigration, electrolytes are conducive to improving metal solubility and can help to overcome the focusing effect by applying a direct current electric field [10,11]. In previous studies, electrolytes containing chelators, surfactants, and other agents were usually used to increase the efficiency of the removal of contaminants [12,13,14]. Table S1 summarizes some operating conditions for EKR taking Zn and other heavy metals as an example [7,8,15,16,17,18,19,20,21]. In laboratory research, the electrolyte chamber is always situated on the ends of the soil chamber. During laboratory tests, the electrodes are usually put into the electrolyte chamber, rather than inserting them into the soil. However, for field applications, the electrolytes are only injected directly into the soil at present, which leads to a high and immediate threat of aqueous contamination plumes in groundwater resources [1,7,8,9]. Moreover, this mode increased the volume of electrolytes for vertical and horizontal penetration in the soil and serious leakage may occur when it encounters sand layers. The contamination of multiple environmental matrices has impeded the overall progress towards attaining sustainable development goals (SDGs) [22].

From an engineering point of view, instead of injecting the electrolytes into the soil, novel environment-friendly electrolyte usage patterns need to be developed. Inspired by the testing apparatus of electroosmosis, Reuss conducted the first in-depth study in 1802 [23], and a novel electrolyte chamber configuration was developed intending to promote the EKR’s sustainability and potential to be scaled up for field applications. Open-ended circular tubes were used as electrolyte chambers. These tubes were partially vertically inserted into the soil. The electrolytes were poured into the unburied part of the electrolyte chambers. The novelty and originality of the EKR configuration was the electrolyte chamber situated above the soil surface. Hence, compared with previous studies, the electrolytes had a smaller contact area with the soil, and this generated weak osmosis in fine-grained soil with low permeability under the force of gravity. Moreover, the ions in the electrolytes can migrate into the soil and enhance heavy metal solubility or transfer heavy metals to the electrolyte chamber through chelation under an electric field. The contaminants transferred to the electrolyte chambers were easy to deal with. Secondary pollution and the pouring of a large amount of electrolyte have been major problems in previous studies. However, this novel configuration can alleviate secondary pollution and significantly reduce the amount of electrolyte pouring into the soil. Furthermore, the novel EKR configuration is cost-effective and eco-friendly, which are important attributes of the sustainability framework. 

Process parameters such as the electrolyte type, concentration, pH, and retention time are crucial for metal desorption/dissolution in the soil [24,25]. Using citric acid (CA), ethylene diamine tetra acetic acid (EDTA), and fulvic acid (FA) to remove heavy metals has proven to be an effective method [13,26,27]. Organic acids such as CA and FA are easily obtained, cheap and effective for most heavy metals with little ecological risk [4]. EDTA has a strong complexing ability and has proven to be a suitable chelating agent for the remediation of heavy metals. However, when using the novel configuration, the performance of these electrolytes in heavy metal removal from fine-grained soil is not clear. 

The present paper seeks to verify the electrokinetic remediation effect of Zn-polluted soft clay using the proposed novel electrolyte chamber configuration. The feasibility of CA, EDTA, and FA as a means to achieve high removal efficiency was further investigated. The Zn content in both the electrolyte and the soil after treatment was tested. Moreover, the drainage reduction effect was also evaluated by testing the soil moisture content, pH value, etc.

## 2. Materials and Methods

### 2.1. Chemicals

Perchloric acid, hydrochloric acid, and sulfuric acid of reagent grade used to digest soil samples was provided by the Analysis and Testing Centre of Nantong University. Other chemicals were purchased from Sinopharm Chemical Reagent Co., Ltd. (China) and were of analytical grade. Deionized distilled water was used in all the experiments.

### 2.2. Simulated Zn-Contaminated Soft Clay

Clay powder was obtained from a clay processing plant in Nanjing, China. The properties of the clay soil are summarized in Table 1. The initial Zn concentration in clay powder was 44 mg/kg. Clay powder was weighed first and the volume of distilled water required to reach a water content of 55% was measured and poured into a container. The mass of zinc sulphate (ZnSO_4_ • 7H_2_O) required for a concentration of about 2000 or 3000 mg [28,29] of zinc per kg of dry soil was then weighed and added to the water container. The soil specimen was prepared by thoroughly mixing the clay powder with the zinc solution using a mechanical stirrer to ensure homogeneous dispersion of Zn. After that, the soil was sealed and placed in a no-light environment for a week to allow for zinc adsorption by soil solids to take place and reach equilibrium. The configured Zn-polluted soft clay was loaded into the EKR cell for each test. 

### 2.3. Novel EKR Experiments

**Figure 1:**
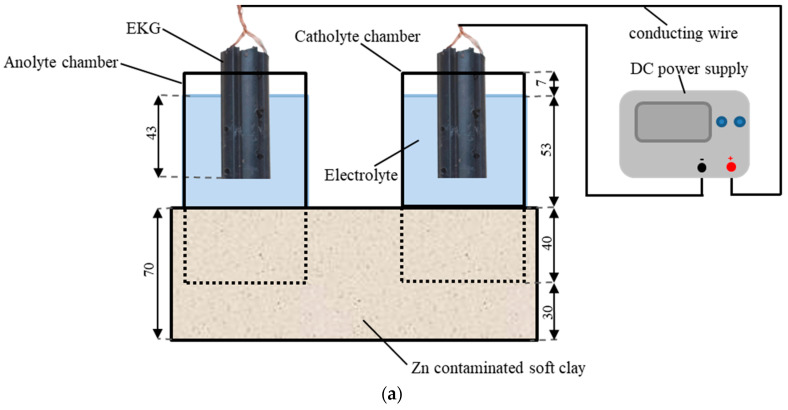

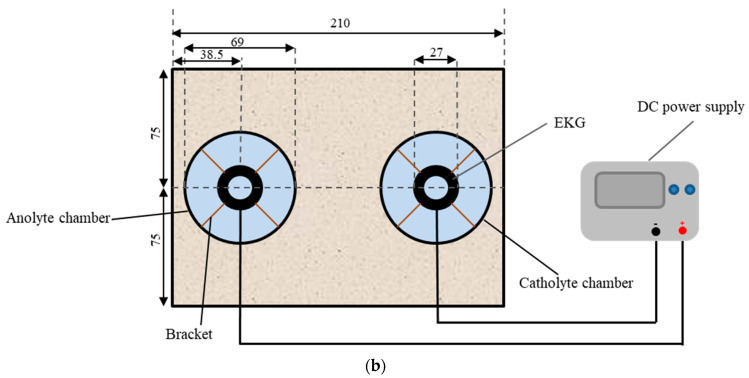
Schematic diagram of the novel electrolyte chamber configuration for electrokinetic remediation: (**a**) front view and (**b**) top view (unit mm).

The EKR cell was made of polymethyl methacrylate with internal dimensions of 210 mm × 150 mm × 70 mm (length × width × height). The cell was filled with simulated Zn-contaminated soft clay. Two electrolyte chambers were vertically inserted into the contaminated soil with a buried depth of 40 mm, as shown in Figure 1a. The electrolyte chamber was an open-ended circular tube with an inner diameter and a height of 69 mm and 100 mm, respectively. The unburied length of the tube electrolyte chamber was 60 mm. Electrokinetic geosynthetics (EKG) were chosen as the electrodes. The EKG were made of polyethylene, carbon black, and graphite with good conductivity and corrosion resistance. Two clusters of copper wires with a diameter of 1 mm were buried in the pipe along the longitudinal direction, as shown in Figure 1a. Details of the EKG have been described by Sun et al. [30]. An amount of 200 mL electrolyte was slowly injected into each of the electrolyte chambers with a height of 53 mm. Due to the low permeability of soft clay, the leakage of electrolytes will not happen. The EKG were put into the unburied section of the electrolyte chambers, without coming into contact with the soil. The EKG were submerged to a depth of 43 mm into the electrolyte and was fixed to the electrolyte chamber by brackets. Conducting wires were used as connections between the direct current power supply (RXN-605D) and the EKG. The plane layout of the novel EKR experiment is shown in Figure 1b.

### 2.4. Test Schemes

The major problem for EKR is how to promote the desorption of Zn from the soil and release it into the liquid phase. Seven series of tests were carried out using the novel EKR configuration, corresponding to the scenarios described in Table 2. Distilled water, CA, EDTA, and FA were selected as the electrolyte for comparison of their remediation efficiency. The electrolyte concentrations of CA, Na2-EDTA, and FA used in this study were 0.2 mol/L, 0.1 mol/L, and 0.05 mol/L, respectively. According to previous studies [31,32,33], the aforementioned concentrations were generally more effective in removing heavy metals from soils. Each 200 mL of electrolyte was poured into the corresponding electrode chambers. The applied voltage during regular working daytime hours was 20 V.

FT1–FT4 were conducted for only 9 h each, with the aim of studying the feasibility of the novel electrolyte chamber configuration. Moreover, the optimal remediation efficiency of the anolyte and catholyte with different compositions was compared. The control group was the anolyte and catholyte for FT1 using distilled water. The catholyte for FT2–FT4 was all CA, their anolyte was distilled water, CA, and NaCl solution (1.76 mol/L), respectively. The initial Zn concentration of FT1–FT4 per kg of dry soil was 1983 mg. 

After that, the remediation efficiency of EDTA, CA, and FA was evaluated by T5–T7. The initial Zn concentration of T5–T7 per kg of dry soil was 3175 mg. The anolyte and catholyte were refreshed every 9 h (daily working hours). The variation in current in the soil of each test was directly read from the DC power supply and recorded. The Zn concentration and pH of the replaced anolyte and catholyte were tested every time. When the Zn concentration in both electrolytes became less than 5 mg/L, the EKR treatment was terminated. 

At the end of each test, the soil was extruded from the cell and divided into three equal sections, S1, S2, and S3, as shown in Figure S1. The water content, pH, and Zn concentration were determined for each section. The Zn concentration was determined using an atomic absorption spectrophotometer (AAS, TAS-990F). To determine the pH and Zn concentration for each of the three sections, a soil sample was air-dried and then ground. Soil pH was determined in accordance with ASTM D4972-13. To determine the Zn concentration, about 0.15 g of dry ground soil was mixed with 5 mL of mixed acids of HCl–HNO_3_–HClO_4_. The mixture was then heated on a hot plate at 200 °C. The concentration of Zn in the supernatant was then determined using AAS. 

## 3. Results and Discussion

### 3.1. pH in Electrolytes and Dry Soil

The initial pH of CA with a concentration of 0.2 mol/L was 3.0, while that of distilled water and NaCl solution was 7.0. During the electrokinetic process, electroosmotic flow and electromigration occurred in the soil, along with electrolysis reactions of water at the electrodes. The reactions resulted in oxidation at the anode generating an acid front and reduction at the cathode producing a base front. Figure S2 illustrates the anolyte and the catholyte pH value variations for the feasibility tests. It was found that the pH in the anolyte decreased for each test while it increased in the catholyte. After 9 h of electrokinetic remediation treatment, the pH in the anolytes decreased to 1. The pH in the catholyte of FT1 increased to 13.0. The highly alkaline environment created favourable conditions for the precipitation of heavy metals. Despite using CA as catholyte for FT2, FT3, and FT4, the pH of FT2 and FT4 increased to 6.0, while that of FT3 increased slightly to 3.5. Hence, all the anolytes and catholytes which used CA were an excellent choice for pH control. 

The electrokinetic remediation treatment times of T5, T6, and T7 were 17 d, 45 d, and 16 d, respectively. The electrolytes were refreshed after every 9 daily working hours. The initial pHs of the EDTA, CA, and FA solutions were 4.5, 2.5, and 4.5, respectively. Before the electrolytes were collected into test tubes after each day’s working hours, the final pH was tested, as shown in Figure S3. The daily final pH in the EDTA anolyte of T5 decreased from 4.5 and then fluctuated between 3.5 and 4.0, that of T6 decreased from 2.5 and then fluctuated between 1.5 and 2.0, and that of T7 decreased from 4.5 and then fluctuated between 2.0 and 2.5. The daily final pH in the EDTA catholyte of T5 rose from 6.5 to 8.5 and then decreased to 6. The CA catholyte of T6 induced favourable pH conditions that were constant at 2.5 and then slightly increased to 3.5, while the FA catholyte of T7 turned to an alkaline environment with a pH range of 11 to 12.5. The electrolyte pH had an influence on the soil pH. Much of the Zn in the solution was in free ionic form when the soil solution pH was below 6.5, while precipitation formed when the pH was between 6.5 and 8 [34]. When the soil pH near the catholyte chamber ranged from 6.5 to 8, Zn accumulated in the soil as precipitate and no Zn could be collected in the catholyte. 

**Figure 2:**
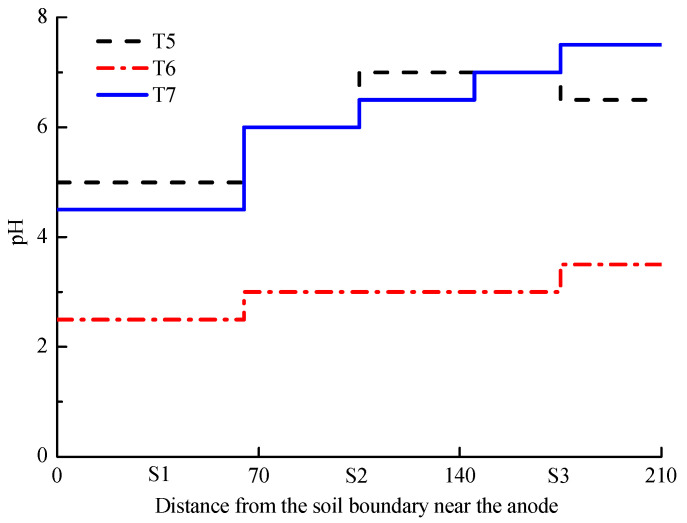
Dry soil pH after EKR treatment of T5–T7.

The initial pH of dry soil before EKR treatment was 6.5. Figure 2 shows the T5–T7 pH results of the dry soil after EKR treatment. The pH values in T5 and T7 were almost identical except in S3 where the pH in T5 decreased from 7 to 6.5. In sections S2 and S3 of T5 and T7, the pH values appeared larger than 6.5, which induced the precipitation of Zn as hydroxide or carbonate [34]. The lower pH in three sections of T6 was favourable for the remediation process since it promoted more dissolution of Zn from the soil matrix into the pore fluid. The electroosmotic flow coupled with electromigration removed the dissolved Zn further towards the cathode. Thus, all the anolyte and catholyte which used the CA solution were capable of overcoming the base front and were beneficial to the soil pH control. 

### 3.2. Heavy Metal Concentration

**Figure 3:**
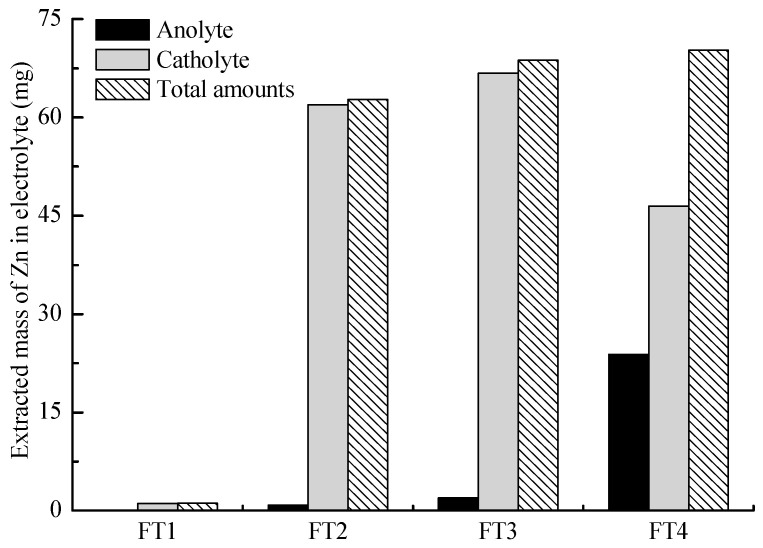
Amounts of Zn extracted in the electrolytes of FT1–FT4 during 9 h electrokinetic treatment.

Amounts of Zn extracted from the electrolytes in the feasibility tests FT1–FT4 after 9 h of electrokinetic treatments are presented in Figure 3. As shown in the figure, 0.1, 0.8, 2.0, and 23.8 mg of Zn migrated into the anolyte of FT1, FT2, FT3, and FT4, respectively, while 1.1, 62.0, 66.8, and 46.5 mg of Zn migrated into the catholyte of FT1, FT2, FT3, and FT4, respectively. Zinc ions in the soil were transported towards the catholyte via electromigration and electroosmotic flow under a direct current field. However, the pH variation in both the soil and electrolytes influenced the morphology of Zn. The catholyte of FT1 was always in an alkaline environment, which induced the zinc ions transported here to react with the hydroxide ions forming zinc hydroxide precipitations. As the pH value continued to increase, zinc hydroxide reacted with hydroxide ions forming [Zn(OH)_3_]^−^ and [Zn(OH)_4_]^2−^ [35], which were transported towards the anolyte via electromigration forming zinc hydroxide precipitations in the soil. Hence, FT1 during 9 h electrokinetic treatment had very low levels of accumulated mass of Zn transported towards the anolyte and catholyte that could be tested by ASS. 

When the catholyte was changed to CA in FT2, the extracted mass of Zn in the catholyte significantly increased. In comparison to FT2, FT3 had a slightly higher accumulated mass of Zn transported towards the anolyte and catholyte. The total FT4 had the highest amount of Zn extracted in the electrolyte; this was due to the use of NaCl solution which greatly promoted the electroosmotic flow towards the catholyte and increased the electric current in the soil. The enhanced electroosmotic flow and electromigration caused more zinc ions to be transported to the CA catholyte. In a tube containing zinc ions and CA the following equilibrium equations are possible:

Zn^2+^ + Cit^3−^ + H^+^ = ZnCitH (1)

2Zn^2+^ + Cit^3−^ − H^+^ = Zn_2_CitH (2)

2Zn^2+^ + 2Cit^3−^ −2H^+^ = Zn_2_Cit_2_H^4−^ (3)

2Zn^2+^ + 2Cit^3−^ − H^+^ = Zn_2_Cit_2_H^3−^ (4)

2Zn^2+^ + 2Cit^3−^ = Zn_2_Cit^2−^ (5)

Zn_2_Cit_2_H^4−^, Zn_2_Cit_2_H^3−^, and Zn_2_Cit^2−^ will be transported towards the anolyte via electromigration. Hence the extracted mass of Zn in the anolyte was higher than that of other feasibility tests. Electrolytes containing chloride ions may cause adverse effects or secondary pollution, hence it may be difficult to apply them on a commercial scale. From an engineering point of view, citric acid may be a better electrolyte, because it is easily obtained, cheap, and effective for most heavy metals with low ecological risk [4]. In summary, the tests performed in this feasibility study indicated that the novel electrolyte chamber configuration to remediate heavy-metal-contaminated soil is promising.

**Figure 4:**
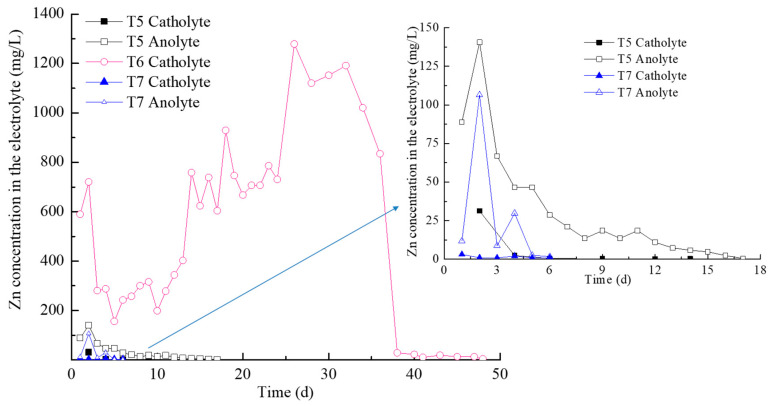
Zn concentration in the electrolytes of T5, T6, and T7.

For T5, T6, and T7 the Zn concentration variations in the electrolytes are shown in Figure 4. The Zn concentration in the catholytes of T6 was significantly higher than in other electrolytes. The electrolyte of T6 was refreshed on a daily basis for 24 days and then refreshed after every two days, while the latter Zn concentration did not increase twice as much as the former. In this context, the electrolytes have to be refreshed timely because superfluous zinc ions in the catholytes formed counter-gradient diffusion. Zn concentration in the anolyte of T5 and T7 increased first and then decreased, and was obviously higher than that in their catholytes. This was because EDTA and FA formed negatively charged complexes with Zn. The negative complex would be transported towards the anode by electromigration when the soil pH is lower than 6.5.

For T5–T7, the amounts of Zn extracted from the electrolytes (m_e_) and the removal efficiency in the electrolytes were calculated by m_e_/m_0_ (m_0_ is the initial Zn mass in the soil) as presented in Table 3, while the Zn removal efficiency in the soil sections is presented in Table 4. The results showed that the removal efficiency calculated by m_e_/m_0_ and 1-c/c_0_ (c is the Zn concentration in the soil at the end of the EKR test and c_0_ is the initial Zn concentration in the soil) were almost consistent for T6 and vastly different for T5 and T7, which resulted in different mass balance errors. In T6, most of the zinc was removed from three soil sections, migrated into the catholytes, and existed in ionic morphology, which was directly detected by AAS. Hence, the mass balance error for T6 was only 1.5 ± 0.6%. Although T5–T7 used the same testing method for the Zn concentration of the soil and electrolytes, the mass balance errors for T5 and T7 were 36.8 ± 0.7% and 66.4 ± 0.5%, respectively. That was mainly because there was some flocculent precipitation suspended in the electrolytes of T5 and T7 at high pH, which was filtered out through a 0.22-μm filter before AAS testing. These Zn masses in the flocculent precipitation were not taken into account, thus causing a mass imbalance of Zn for T5 and T7. In T7, the removal efficiency of S1 was 94.7 ± 0.2% and it decreased from S1 to S3. In T5 most of the zinc was removed from S1 near the anode as well as S3 near the cathode. However, more zinc accumulated in S2, due to many complication factors, such as pH variation, the low mobility of EDTA, and the contradicting directions of Zn^2+^ cation and negatively charged EDTA–Zn complexes [36]. 

### 3.3. Electric Current

**Figure 5:**
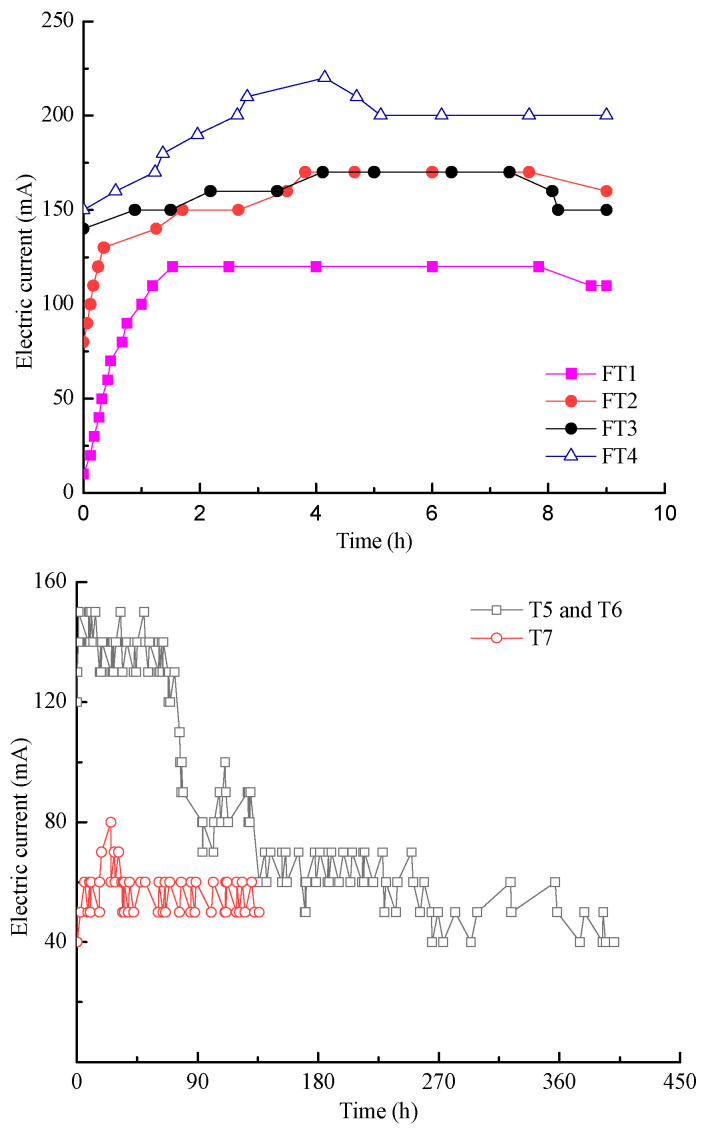
Electric current variation during EKR treatment.

The magnitude of the electric current is the charges flowing through the cross-section of the pore liquid per unit of time. These charges are the sum of the simultaneously migrating cationic and anionic charges. Figure 5 shows the electric current variations for each test during EKR treatment. The electric current of feasibility tests increased first and then stabilized, finally showing a downward trend. The introduction of Na^+^ in FT4 significantly increased the electric current and it was higher than in other tests. The changes in the electric current trend for FT2 and FT3 were similar and higher than that of FT1. Compared to FT1, the CA catholyte in FT2 and FT3 introduced H^+^ and reduced the formation of precipitation in the soil. When the treatment time was increased, the electric current of T5 and T6 decreased gradually because the continuous daily removal of zinc in the soil reduced the soil conductivity, while that of T7 increased first to 80 mA and then fluctuated between 50 and 60 mA. The decrease in the electric current in the heavy-metal-contaminated soil was mostly associated with the removal or precipitation of heavy metals in the soil, which reduced the conductivity of the soil. 

### 3.4. Water Content 

**Figure 6:**
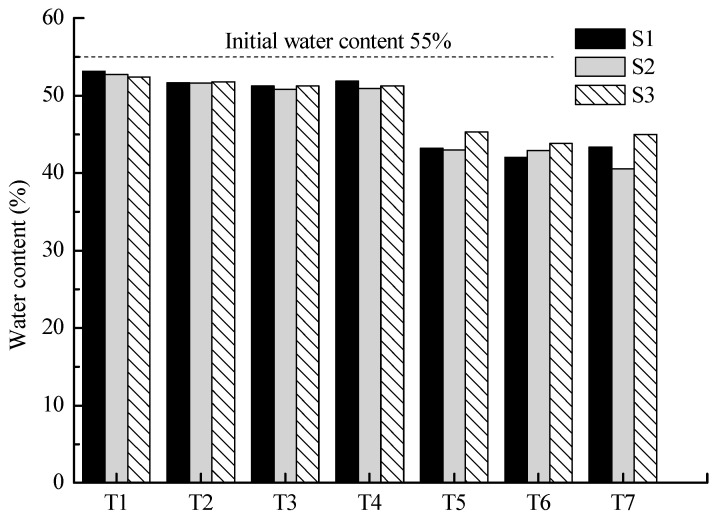
Soil water content after EKR treatment.

The water content of soil sections after tests is shown in Figure 6. The difference in the water content in S1, S2, and S3 of each test was not significant (the maximum difference is 4.4% between S1 and S2 in T7) as seen in the figure; this indicates that the distribution of water content in the soil after EKR treatment was relatively even. The average water content in the four feasibility tests was close, with a range of 51% to 52%, indicating that the electrolyte composition had little influence on the soil water content distribution. The average water content of T5, T6, and T7 with different processing times and electrolyte compositions was also about 43%, which represents the limiting water content after the novel EKR treatment. The water content in the soil decreased with an increase in EKR processing time. However, the supplementing of the electrolytes resulted in the water content being distributed evenly in the soil and finally sustained at approximately 43%. 

## 4. Conclusions

In this study, experiments were conducted to evaluate the applicability of the novel EKR configuration and the effect of different electrolyte compositions on the electrokinetic remedial efficiency. The success of the novel EKR configuration in removing metal Zn depends on the choice of the facilitating agents. All the anolytes and catholytes which used 0.2 M CA were an excellent choice for pH control for both the soil and the electrolytes. The removal efficiency was relatively uniform in different soil sections and more than 90% of the initial Zn was removed. However, from the anode to the cathode the removal efficiency of Zn in the soil decreased from 94.7 ± 0.2% to 34.3 ± 0.8% when FA was used as the electrolyte. When EDTA was used as the electrolyte, more zinc accumulated in the middle section of the soil. The optimal mass balance errors of Zn were 1.5 ± 0.6 % when CA was used as the electrolyte, indicating that the removal efficiency of Zn in the soil can be directly determined according to the Zn concentration in the electrolytes. The addition of sodium in the anolyte enhanced the electric current and electroosmotic flow, which was conducive to the removal of zinc in the soil, and the removal or precipitation of heavy metals in the soil decreased the electric current. The water content in the soil decreased with an increase in EKR processing time. However, the supplementing of the electrolytes resulted in the water content being distributed evenly in the soil and finally sustained at approximately 43%. The proposed novel electrolyte chamber configuration is feasible for heavy-metal-contaminated fine-grained soil, reducing the overall amount of electrolyte used, and conducive to reducing the leakage of electrolyte solution and alleviating secondary pollution, finally promoting the EKR potential to be scaled up for application. The novel EKR configuration is cost-effective and eco-friendly among other benefits to society.

## Figures and Tables

**Table 1:** Characteristics of clay powder used in this study.

Properties	Value
Particle size analysis	
Fine sand (%)	15
Silt (%)	49
Clay (%)	36
Water content (%)	6
pH	6.5
Liquid limit (%)	43
Plastic limit (%)	23
Zn (mg/kg)	44

**Table 2:** Experimental scenario for EKR tests.

Test Number	Anolyte Solution	Catholyte Solution	Electrolyte Refreshed Time ^a^	Initial Zn Concentration (mg/kg)	Total Processing Time ^b^	Initial Soil Moisture Content (%)	Initial Electrolyte Volume for Each Electrode Chamber (mL)	Applied Voltage (V)
FT1	Distilled water	Distilled water	/	1983.2 ± 1.4	1 day	55.0 ± 0.5	200	20
FT2	Distilled water	0.2 M CA						
FT3	0.2 M CA	0.2 M CA						
FT4	1.76 M NaCl	0.2 M CA						
T5	0.1 M EDTA	0.1 M EDTA	1 day	3175.4 ± 2.7	17 days			
T6	0.2 M CA	0.2 M CA	1 day or 2 days		48 days			
T7	0.05 M FA	0.05 M FA	1 day		6 days			

^a^ Total daily working hours were 9 h. ^b^ The total processing time for T5–T7 was determined according to Zn concentration in the electrolytes.

**Table 3:** Extracted mass of Zn from electrolytes and removal efficiency for T5, T6, and T7.

Test Number	Extracted Mass of Zn in Electrolytes m_e_ (mg)	Initial Zn Mass in Soil m_0_ (mg)	Removal Efficiency (m_e_/m_0_) %
T5	99.9 ± 7.6	7169.2 ± 12.9	1.4 ± 0.1
T6	6667.2 ± 11.8		93.0 ± 0.0
T7	56.3 ± 6.3		0.8 ± 0.1

**Table 4:** Zn removal efficiency in the soil sections and mass balance for T5, T6, and T7.

Test Number	Soil Removal Efficiency (1-c/c_0_) %				Mass of Zn in the Soil (%)	Mass of Zn in the Electrolyte (%)	Error (%)
	S1	S2	S3	Average			
T5	78.9 ± 0.3	−28.3 ± 0.1	64.1 ± 1.4	38.2 ± 0.6	61.8 ± 0.6	1.4 ± 0.1	36.8 ± 0.7
T6	97.2 ± 0.2	96.4 ± 1.1	89.8 ± 0.5	94.5 ± 0.6	5.5 ± 0.6	93.0 ± 0.0	1.5 ± 0.6
T7	94.7 ± 0.2	72.7 ± 0.3	34.3 ± 0.8	67.2 ± 0.4	32.8 ± 0.4	0.8 ± 0.1	66.4 ± 0.5

## Data Availability

Not applicable.

## Supplementary Materials

The supporting information can be downloaded at: https://www.mdpi.com/article/10.3390/toxics11030263/s1.
