# Supplementary material for: Electrokinetic Remediation of Zn-Polluted Soft Clay Using a Novel Electrolyte Chamber Configuration
Source: Toxics. 2023 Mar 12;11(3):263. doi: 10.3390/toxics11030263 (PMC10051708; doi:10.3390/toxics11030263)
Supplement: Supplementary file 1 [file toxics-11-00263-s001.zip › toxics-2225299-supplementary.pdf]

toxics

MDPI

Table S1. Operating conditions for EKR of Zn and other heavy metals.

Soil type	Research method	Anolyte	Catholyte	Electrolyte location	Best removal rate of Zn (other heavy metals) (%)	Reference
A dredged sediment sample collected at the disposal site	Laboratory model test	0.1 M EDDS<br>0.1 M CA	0.1 M EDDS<br>0.1 M CA	The ends of the soil chamber	41.4	(Ammami et al., 2022)
Railroad soil contaminated by lubricant oil and Zn	Laboratory model test	0.1 M $MgSO_4$ + 0.5 wt% tergitol	0.1 M HNO_3	The ends of the soil chamber	24.3	(Park et al. 2009)
Pb, Zn, and Cu contaminated Kaolin using sodium alginate and chitosan as biodegradable complexing agents	Laboratory model test	Distilled water	0.1M HNO_3	The ends of the soil chamber	95.0	(Wang et al., 2021)
Agricultural soil contaminated with multiple heavy metals	Laboratory model test	Deionized water<br>0.1 M NaOH<br>0.5 M CA	Deionized water<br>0.1 M CA<br>0.1 M EDTA<br>0.5 M CA	The ends of the soil chamber	73.3	(Cameselle et al., 2021)
Dewatered sludge (approaching anode technique)	Laboratory model test	Deionized water<br>0.1 M EDDS	Deionized water<br>0.1 M EDDS	The ends of the soil chamber	56.8	(Tang et al., 2020)
Calcareous soil contaminated with Zn, Cd and Pb (anode displacement system)	Laboratory model test	0.01 M acetic acid<br>EDTA	0.01 M acetic acid<br>EDTA	The ends of the soil chamber	40.1	(Beyrami 2021)
Wast landfill site soil contaminated with Cu	Field test	Water + 0.05 M CA	The perforated cathode pile filled with reactive materials	Slotted perforated PVC used as the electrolyte chamber and installed vertically to a depth of 4 m below the ground surface	85.2 (Cu)	(Chung 2009)
A galvanizing plant site in Delft	Field test	Electrode solution at pH 4-5	-	Anode locations installed up to 1 m below ground surface	33.0	(Lageman, 1993)
A rice field near a zinc refinery plant	Field test	NaOH	Water<br>EDTA	Anode and cathode locations installed up to 1.5 m below ground surface	17.2 (Cu)<br>39.8 (As)<br>19.4 (Pb)	(Kim et al. 2012)

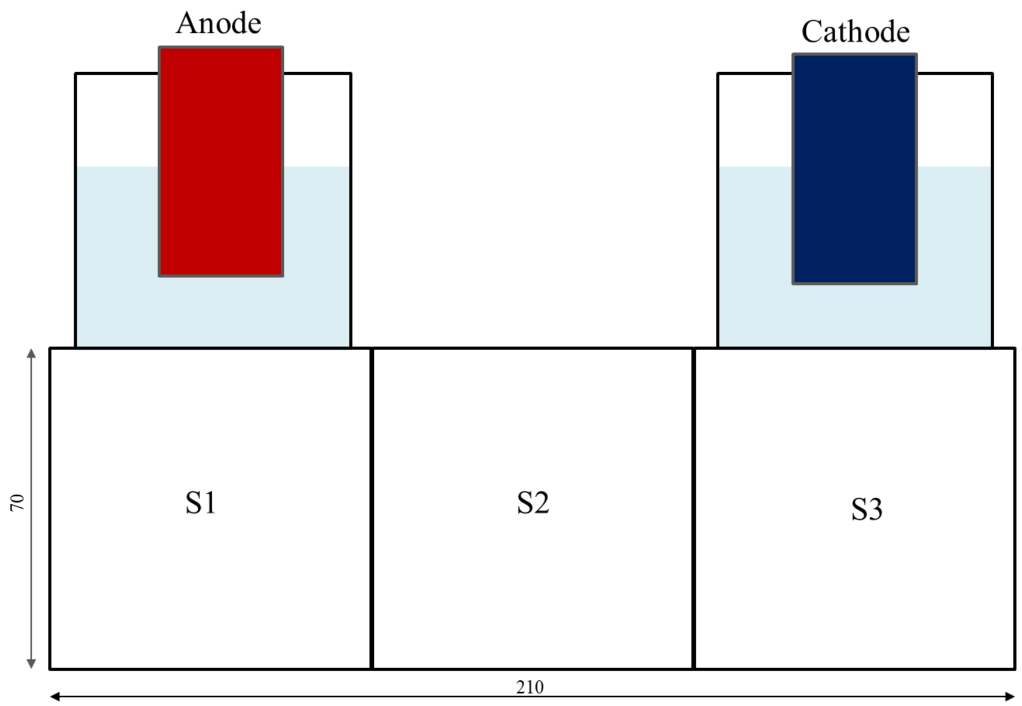

Figure S1. Soil sections represented as S1-S3 for water content, pH, and Zn concentration analysis (unit mm).

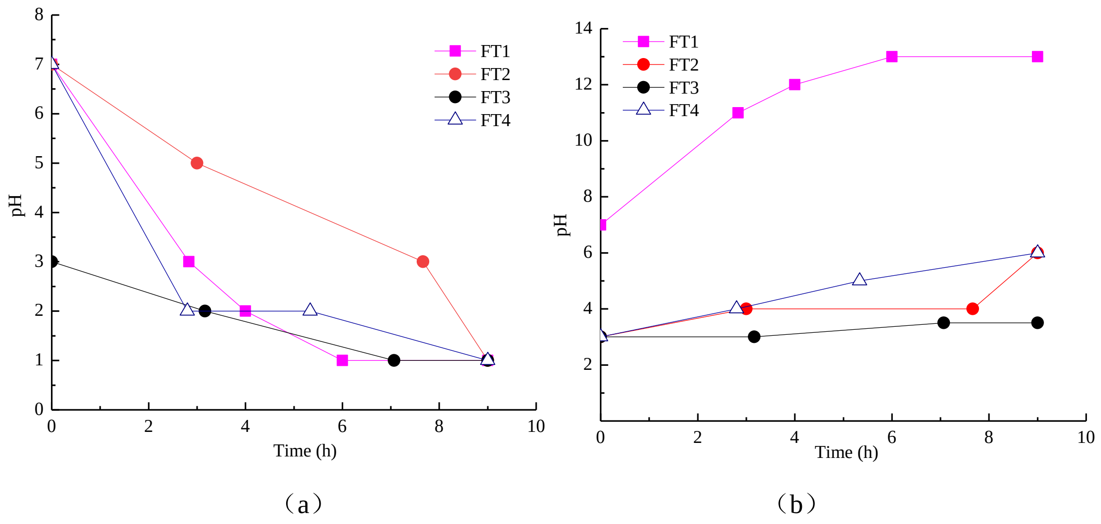

Figure S2. pH in electrolytes of feasibility tests: (a) anolyte and (b) catholyte.

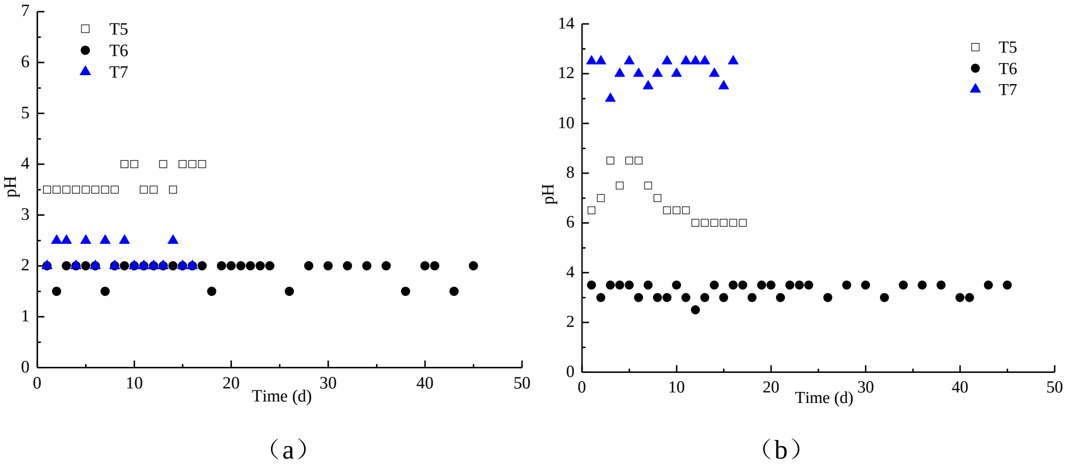

Figure S3. pH in electrolytes of T5, T6, and T7 (a) anolyte and (b) catholyte.
